# What’s missing in autism spectrum disorder motor assessments?

**DOI:** 10.1186/s11689-018-9257-6

**Published:** 2018-12-13

**Authors:** Rujuta B. Wilson, James T. McCracken, Nicole J. Rinehart, Shafali S. Jeste

**Affiliations:** 1 UCLA Semel Institute of Neuroscience and Human Behavior, David Geffen School of Medicine, 760 Westwood Plaza, Room A7-424, Los Angeles, CA 90095 USA; 2 Deakin University, Deakin Child Study Centre, School of Psychology, Faculty of Health, 221 Burwood Highway, Burwood, Geelong, VIC 3125 Australia

**Keywords:** Motor function, Autism spectrum disorder, Motor assessments, Quantitative motor measures

## Abstract

**Background:**

Motor delays and impairments in autism spectrum disorders (ASD) are extremely common and often herald the emergence of pervasive atypical development. Clinical accounts of ASD and standardized measures of motor function have identified deficits in multiple motor domains. However, literature describing frequently used standardized motor assessments in children with ASD, their test properties, and their limitations are sparse.

**Methods:**

We systematically reviewed the literature to identify the most frequently used standardized motor assessments used to evaluate children with ASD from infancy to early childhood. All assessments included were required to possess reference norms, evaluate more than one motor domain, and have undergone some degree of validation.

**Results:**

We identified six frequently used standardized measures of motor function per our inclusion and exclusion criteria. We investigated and described in detail the psychometric properties of these assessments, their utility for use with children with ASD, and their individual and overall strengths and limitations. The global strengths of these assessments are the ability to identify early development delays and differences in fine and gross motor function in children with ASD. Global limitations of these studies are lack of validation in individuals with ASD and scoring systems that often miss specific and subtle abnormalities.

**Conclusions:**

Standardized assessments of motor function have provided valuable information on motor impairments in ASD. However, significant limitations remain in the use of these measures in children with ASD. Moving forward, it is imperative that standardized measures of motor function receive greater validation testing in children with ASD to assess their potential application given the clinical heterogeneity of this condition. In addition, utilizing quantitative measures of motor function should allow for evaluation and comparison of individuals with ASD across the lifespan with varying cognitive and behavioral abilities.

## Background

Autism spectrum disorder (ASD) represents a heterogeneous and complex group of neurodevelopmental disorders defined by core deficits in social communication, the presence of restrictive and repetitive behaviors, and unusual sensory sensitivity. While not a core diagnostic feature, motor delays and abnormalities are present in the majority of children with ASD [1, 2]. Motor abnormalities in ASD manifest early and often predate the emergence of canonical core deficits of ASD. Motor problems are also intrinsically linked to core features of ASD such as language and adaptive functioning [3–5]. Moreover, motor function is a common intervention target, thus accurate measurement to individualize therapy can improve overall outcomes.

Early descriptions of ASD highlighted the co-occurrence of motor abnormalities with its core features, such as clumsy gait, odd posture, and ill coordination in these children [6, 7]. Since then, various measures have been utilized to capture multiple motor domains affected in ASD including movement accuracy, reaction time, fine and gross motor, gait, balance stability, hyperkinesis, and praxis [4]. There have also been rapid developments in the identification of genetic risk factors for ASD that better define the underlying mechanisms of the disorder [8]. These genetic syndromes that are highly penetrant for ASD often present with prominent motor delays even prior to identification of core symptoms of ASD [9, 10]. Despite the pervasive and variable nature of motor abnormalities in ASD and their importance, the standardization and guidelines for motor phenotyping lack uniformity and have lagged behind that of other behavioral and developmental measures.

Available standardized measures capture motor milestones and skill acquisition but they often do not capture more qualitative or subtle differences in overall motor function. As with all other neurodevelopmental domains (such as cognition or social skills), a better assessment of the full spectrum of differences and impairments in motor skills would serve two key purposes: shed light on specific neural mechanisms of atypical development and provide more specific targets for motor-based interventions which, in theory, could drive improvement in other core features of ASD. For example, rather than quantifying whether a child is able to walk from point A to point B, it would be more informative to evaluate if the gait is wide based, rigid, or asymmetric which suggest different underlying mechanisms that might be implicated.

Here we first systematically review the literature describing the most frequently used standardized assessments of motor function in ASD from infancy to early childhood, detailing domains examined, the overall information provided, and major strengths and limitations of the assessments based on the opinion of the authors and review of the literature. We then provide specific suggestions on next steps in the assessment of motor skills in ASD, with focus on qualitative and quantitative evaluation of motor function to better assess specific motor domains.

## Methods

To identify standardized motor assessments in ASD, a PubMed search was performed with the key search terms “motor” and “autism.” The initial search identified 2210 records. From these articles, standardized motor assessments were selected for inclusion if they met all of the following six predetermined criteria:

1. Assessments must include a direct motor examination (caregiver report is insufficient).
2. Assessments must include children between age of birth and 4 years.
3. Assessments must be used in children with a diagnosis of ASD or ASD high-risk status.
4. Assessments must be norm referenced and validated.
5. Assessments must include evaluation of at least fine motor *and* gross motor domains.
6. Assessments that fit the above criteria must be described in two or more published studies.

### Methods for assessment details

The descriptors of interest for the standardized motor assessments were chosen to provide the reader with a comprehensive overview of each assessment. Descriptors included the age range of participants, time to complete the assessment, motor domains tested, the scoring system, psychometric properties of the assessment (specifically, the reliability and validity of the test and the normative sample used for standardization), and their use in special populations. For each assessment, an additional search was performed in PubMed to obtain the original article and any supporting articles to complete the table.

## Results

Six standard motor assessments were identified. Reference lists of articles that described one of the six assessments were also reviewed for other motor assessments. No additional assessments were identified. The six standardized motor assessments include (1) the Mullen Scales of Early Learning (MSEL), (2) the Bayley Scales of Infant and Toddler Development-III (Bayley-III), (3) the Peabody Developmental Motor Scale-2 (PDMS-2), (4) the Movement Assessment Battery for Children-2 (MABC-2), (5) the Bruininks–Oseretsky Test of Motor Proficiency-2 (BOT-2), and (6) the Physical and Neurological Examination for Soft Signs (PANESS). Of potential interest to the reader, other motor assessments used frequently in individuals with ASD, but did not meet inclusion criteria #5 or #6 for this review include the Alberta Infant Motor Scale, the Test of Gross Motor Development-2, the Beery Test of Visual Motor Integration, and the Zurich Neuromotor Test.

Characteristics of the six assessments are listed in Table 1 and described below. In the descriptions, we have included the individual and overall strengths and limitations of the assessments when evaluating individuals with ASD.

**Table 1:** Detailed characteristics of the six motor assessments

Motor assessment	Age range	Time to complete	Motor domains assessed	Scoring system	Psychometric properties	Special populations:
Peabody Developmental Motor Scale-2 (PDMS-2) [14]	Birth-5 years	45–60 min	*Gross and fine motor* Derived from 6 Subtests: *Reflexes*-reaction to the environment (birth to 11 months) *Stationary*-ability to sustain control of body *Locomotion*-ability to move from one place to another (crawling, walking, running etc.) *Object manipulation*-ability to manipulate balls, *Grasping*-ability to use hands *Visual motor integration*-ability to use visual perceptual skills to perform complex eye-hand coordination tasks. Results of subtests generate three composites: *Gross motor quotient* (GMQ) (reflexes, stationary, locomotion, object manipulation) *Fine motor quotient* (FMQ) (grasping, visual-motor integration) *Total Motor Quotient* (TMQ)	*Scored 0–2* 0: will not/cannot attempt item 1: performance shows clear resemblance to mastery criteria but does not fully meet criteria 2: child performs item according to criteria specified for mastery Basal level: score of 2 on three items in a row Ceiling level: score of 0 on three items in a row	Normative sample collected in 1998 on 2003 kids in 46 states in the United States (US) and one Canadian province. Normative sample matched U.S. Bureau of the Census in 1997 with regard to geographic region, gender, race, residence, ethnicity, and socioeconomic status. Inter-rater reliability: GMQ (*r* = 0.97), FMQ (*r* = 0.98), TMQ (*r* = .96) Test-retest reliability: GMQ (*r* = 0.93), FMQ (*r* = 0.94), TMQ (*r* = 0.96) Construct validity: Supported using confirmatory factor analysis and reported in manual Concurrent validity With PDMS: GMQ (*r* = 0.84), FMQ(*r* = 0.91) With MSEL: FMQ (*r* = 0.86), FMQ (*r* = 0.80) [14, 43, 44]	22q.11 Deletion [45] Hemi-facial microsomia [46]
Bayley Scales of Infant and Toddler Development-III [13]	1–42 months	50–90 min	*Gross motor*: movement of limb and torso, balance, dynamic movement, static position, motor planning *Fine motor***:** comprehension (grasping), perceptual-motor integration, motor planning and speed, visual tracking, reaching, object grasping, object manipulation, functional hand skills, and responses to tactile information Other Domains: Cognitive, Language, Socio-emotional, and Adaptive	*Scored 0–1* 0: Does not complete 1: Able to complete	Normative sample collected in 2004 on 1700 typically developing children in the US ages 16 days–42 months (born between 36 and 42 weeks) 17 different age groups (*n* = 100 per group). Sample matched the U.S. Bureau of Census in 2000. Sample stratified by age, sex, race/ethnicity, geographic region, and caregiver’s highest education level. 10% of sample: children with mental, physical, and behavioral difficulties. Inter-rater reliability: FM (0.86), GM (0.91) Test-retest reliability: FM (*r* = 0.67), GM (*r* = 0.94), correlations increased with age) Construct validity: Insufficient Concurrent validity: With PDMS-2: FM&GM (*r* = 0.49–0.57)	-Angelman syndrome [47, 48] -Down syndrome [49–51] -Phelan-McDermid (22q13.3 deletion) syndrome [52] -Williams syndrome [53]
Mullen Scales of Early Learning (MSEL) [11]	Gross Motor: Birth-33 months Fine Motor: Birth-68 Months	15 min (1 year); 25–35 min (3 years); 40–60 min (5 years)	*Gross and fine motor* Other subdomains: visual reception, receptive language, expressive language (later four combined to yield the early learning composite)	Varies by item, from 0 to 5 points. Majority of items with 0–1 scoring. 0: correct response 1: incorrect response Basal level: score of 1 on three consecutive items Ceiling level: score of 0 on three consecutive items	Normative sample collected on 1849 children between 2 days and 69 months, grouped into 16 age groups at 2-month age intervals. Sample representative of 1990 US Census data based on gender, race, SES, geographic region, and community size. Children with disabilities were excluded Inter-rater reliability: Gross motor 0–24 months (*r* = 0.92–0.97), fine motor 0–44 months (*r* = 0.91–0.99) Test-retest reliability: Gross motor (*r* = 0.96), fine motor (*r* = 0.83) Construct validity: Confirmed via developmental progression of scores, intercorrelations, and principal factor analysis Concurrent validity with Bayley Scales of Infant Development Gross Motor (*r* = 0.76) Fine Motor (*r* = 0.21). With PDMS fine motor scale 6–36 months (*r* = 0.65–0.75)	-Down syndrome [54] -Fragile X [55–59] -Tuberous sclerosis complex [60–62] -Dup15q syndrome [9] -Williams syndrome [63]
Movement Assessment Battery for Children-2 (MABC-2, age band 1) [16]	3-6 years	20–30 min	*Gross and fine motor* *Manual dexterity*: Posting coins, pegboard, threading bead, bicycle trails *Aiming and catching*: aiming and catching bean bag or tennis ball *Balance*: ability to balance on one leg for 30 s, able to walk on tip toes and tandem	Each item is rated on a 6-point scale 5: weakest performance and 0: best performance. Total test score derived from component scores and translated into percentile rank for each component and overall	Authors assumed reliability and validity data for MABC is generalizable to MABC-2 Normative sample for MABC-2 collected in a group of 1172 children in the United Kingdom (UK) between the ages of 3:0 and 16:11. Sample representative of UK Census in 2001. 48.3% males and 51.7% females Limited information in manual on reliability and validity for age band 1. Inter-rater reliability: Not available for MABC-2 Test-retest reliability: Total of 60 children, 20 from each age band. Total test score (*r* = 0.80) Construct validity: Not available for MABC-2 Concurrent validity: Not available for MABC-2 [16, 18]	-Down syndrome [64, 65] -Turner syndrome [66, 67] -22q11.2 Deletion [68]
Bruininks–Oseretsky Test of Motor Proficiency-2 (BOT-2) [23]	4–21 years	10 min set up, 40–60 min for long form, 5 min set-up, 15–20 min for short form	*Gross and fine motor*: Four Motor Area Composites: *Fine manual control*: control and coordination of the distal musculature of the hands and fingers involved in writing and drawing *Manual coordination*: control and coordination of the arms and hands, especially for object manipulation and with emphasis on speed, and dexterity *Body coordination*: control and coordination of the large musculature used in maintaining posture and balance *Strength and agility*: control and coordination of the large musculature involved in locomotion in sports. Results of composites generate: Total Motor Composite	Scoring varies per item which ranges from a 2- to 13-point scale. Raw scores determined by item and multiple trials when allowed. Raw scores reflect variety of possibilities (i.e., number of correct responses, seconds an activity is sustained, specific directions provided). Subtests and total motor score available as raw score, standard score, percentile rank, and descriptive category (well below average, below average, average, above average, and well above average).	Normative sample of 1520 people 4–21 years old. Stratified across race/ethnicity, socioeconomic status, and disability. Sample representative of 2001 US Census Bureau. Males and females were equally represented. Included children with attention-deficit hyperactivity disorder, emotional and behavioral disturbance, specific learning disability, mental retardation developmental delay, and speech/language impairment. Three additional clinical samples identified for standardization: developmental coordination disorder, high-functioning ASD/Asperger’s, and mild to moderate mental retardation Inter-rater reliability of all subtests: 4–21 years (*r* = 0.84–0.99) Test-retest reliability of all subtests: 4–7 years (*r* = 0.47–0.88), 8–12 years (r = 0.47–0.94), 13–21 years (*r* = 0.32–0.91) Construct validity: Rasch and confirmatory factor analysis Concurrent validity: Total Motor Composite. BOTMP *r* = 0.76) PDMS (*r* = 0.77), Test of Visual Motor Skills and BOT-2 Fine Motor Integration Subtest (*r* = 0.74) [69]	-Down syndrome [70–72] Prader Willi syndrome [73] -Williams syndrome [74] -22q11 deletion [45]
Physical and Neurological Examination for Soft Signs (PANESS) [27]	4–15 years	15–20 min	*Gross and fine motor*: Total of 43 items in subtests: coordination, graphesthesia, stereognosis, motor tasks, ability to maintain posture, ability to tap fingers and feet in a smooth rhythm, string test (visual tracking of moving string) Two subscores generated: Gaits and stations and total times. Subscores are summed to provide PANESS total score. [28, 75]	*Scored 1–4 or 9* 1-Performed correctly 2-Performed not well 3-Performed poorly or after repeated instruction and demonstration 4-Unsuccessful even after repeated demonstrations 9-Not done/not ascertained -Certain tasks are completed using non-preferred and preferred hand and are timed	Normative sample from 168 elementary aged children with average IQ Test-retest reliability: Total score: (*r* = 0.78) -Moderately reliable in a study conducted by Holden et al. Validity: has been established with revised versions [29, 76]	-Neurofibromatosis type 1 [77]

### Assessments

#### Mullen Scales of Early Learning

The MSEL was the most frequently identified assessment of motor skills in children with ASD and genetic conditions associated with ASD. The MSEL also has been widely used to evaluate infants at high risk for ASD. The MSEL includes five different developmental domains (listed in Table 1) of which four span the age of birth to 68 months of age, including the fine motor subscale. However, the gross motor subscale only assesses children through 33 months of age. Most of the items on the fine and gross motor subscales focus on developmental milestones such as the ability to roll, sit unsupported, and use a mature pincer grasp. Nevertheless, in the fine motor section, there are tasks that test multi-step fine motor skills such as stringing beads and screwing and unscrewing nuts and bolts. Evaluation requires 15 min for younger children and up to 60 min for children 5 years of age. The MSEL has shown both concurrent and construct validity, but the normative sample did not include any subgroups of children with developmental delays or ASD [11].

A strength of the MSEL is that the tasks are useful to identify early developmental milestones. The scale has wide utility as well—primary care practitioners, therapists, and caregivers can use the results of the MSEL to supplement their clinical observations of early development and response to intervention. The major limitation of the MSEL is that the majority of the scoring is binary. A child is most often scored as either able to complete or not able to complete a task, which focuses more on skill acquisition rather than providing a scaled score across a range of motor ability.

#### Bayley Scales of Infant and Toddler Development-III

The Bayley-III evaluates children from 1 through 42 months of age thus primarily focusing on infancy through early childhood. Similar to the MSEL, the Bayley-III has been used frequently to assess children with genetic conditions that confer a high risk for ASD. The Bayley evaluates development of preterm infants and thus in the ASD literature has been frequently used to examine the motor profile of preterm infants that later go on to receive an ASD diagnosis [12].

The Bayley-III evaluates six different developmental domains (listed in Table 1). Two of the six domains include fine and gross motor. Although all motor items are grouped under gross or fine motor, there are further descriptors within each domain such as motor planning, visual tracking, and response to tactile information. All items on the Bayley-III are scored as “0” (not able to complete) or “1” (able to complete). The Bayley-III has shown concurrent validity but there is insufficient information to identify the construct validity. Ten percent of the normative sample did include children with atypical development, such as pervasive developmental disorder and Down syndrome [13].

Similar to the MSEL, the Bayley-III identifies and monitors achievement of developmental milestones. The Bayley-III also evaluates in a more granular manner the motor abilities to be tested in certain categories. For example, in the Bayley-III, there is a “grasping series” in which children are noted to be able to grasp with their whole palm, static tripod (thumb and two fingers), or quadrapod (thumb and three fingers) on various different objects [13]. The multi-level depth of the series allows a child to show initial development versus mastery of a skill. Limitations of the Bayley-III is its binary scoring system and no allowance for any range of scoring outside of not able to or able to complete on the motor portions. The Bayley-III is also one of the lengthiest assessments in this review, taking anywhere from 50 to 90 min to complete all domains. Testing may require more than one session and might be difficult for children with ASD to complete if there are significant behavioral co-morbidities.

#### Peabody Developmental Motor Scale-2

The PDMS-2 provides evaluation from birth to 5 years of age. The motor domains are divided into gross and fine motor but unique to the PDMS-II is the reflexes subdomain to evaluate reaction to the environment from birth to 11 months. All items on the PDMS-2 are scored as “0” (will not/cannot attempt item), “1” (performance shows clear resemblance to mastery), and “2” (child performs item according to criteria specified for mastery). Administration time for the test in its entirety is 45–60 min. The PDMS-II normative sample did not include children with disabilities or ASD. The test has shown both concurrent and construct validity [14].

Similar to the MSEL and Bayley-III, a strength of the PDMS includes evaluation of early developmental milestones. However, the PDMS-2 also includes assessment of visual motor integration, which explores integration of motor systems such as visuomotor connections, which have been shown to be potentially aberrant in ASD [15]. Also unique to the PDMS-II is the reflexes category for birth to 11 months of age [14]. Evaluation of reflexes could aid in identifying motor abnormalities in very early infancy and may also help to differentiate ASD from genetic and metabolic conditions that present with abnormal reflexes and delayed motor milestones. The PDMS-II also includes multi-level evaluation of certain skills. For example, the PDMS-II includes a series evaluating a child’s ability to release, grasp, hold, and manipulate a rattle [14]. The PDMS-II does not include a large range to capture subtle differences in motor ability; however, a child can receive partial credit if resemblance of the skill is identified. Compared to the MSEL and Bayley-III, the PDMS-II has not been used as frequently in assessing young children with ASD or genetic conditions that confer a high risk for ASD.

#### Movement Assessment Battery for Children-2

The MABC-2 age band 1 provides evaluation for children 3–6 years of age. Of note, the MABC-2 age bands 2 and 3 span an age range through 16 years but are not covered in this review. In comparison to the BOT-2 and PANESS, the MABC-2 has been used more frequently in the literature to evaluate children with ASD. The assessment consists of eight individual test items measuring fine and gross motor using three categories: manual dexterity, aiming and catching, and balance. The scoring for the MABC-2 is affected by more than the child’s ability or inability to complete a task; thus, for the purposes of this review, we will provide greater detail regarding the scoring mechanism for this assessment. Each item is rated on a 6-point rating scale, where 5 equates weakest performance and 0 equates best performance. Each item is given a raw score and standard score. These scores translate to a component score and percentile for each of the three categories. From the three categories, a total test score is derived and an overall percentile in that child’s age band. If a child has difficulty with a task, the examiner can mark “R” for refusal “I” for inappropriate and “F” for failure. If one or more items are marked as refused or inappropriate, it is not possible to calculate a total test score or the score for any component that has a missing item. The manual dexterity category is composed of posting coins, threading beads, and drawing along a fixed trail. The posting coins category requires the child to complete trials with the preferred and non-preferred hand in order to receive full credit. The posting coins and threading beads tasks are also timed, and scoring is impacted by the length of time a child takes to complete the task. The drawing task does require the child to stay within a predetermined track and scored based on the number of times a child deviates from the line. The aiming and catching category includes catching a beanbag for 10 total trials and throwing the beanbag onto a mat (must hit mat to count as complete) for 10 total trials. In the balance category, the one leg balance is a timed tasked requiring 30 s of balance on the left and right legs. The other two tasks in the balance category are not timed but are scored based off on a required number of trials [16]*.*

The administration time of the assessment is 20–30 min. In regard to the psychometric properties of the MABC-2, the authors assumed that reliability and validity data for the MABC could be generalizable to the MABC-2. The normative sample for the MABC was collected from children in the UK ages 3–16 and did not include children with ASD. There is limited information in the manual on reliability and validity for age band 1. The authors also note that more data is needed to establish test-retest and inter-rater reliability for ages three and four [17, 18]. The MABC-2 shows strength in that the tasks move away from evaluating only developmental milestones and include fine and gross motor skills used in daily activities. The tasks are also meant to engage 3–6-year-old children by including fun items such as throwing and catching. In addition, the MABC-2 allows for some verbal and physical demonstrations to ensure children with lower cognitive ability are better able to grasp the mandatory tasks [19]. However, there are also limitations in the task requirements when evaluating children with ASD. Although minimal demonstrations of a task are allowed, a child needs the cognitive ability to understand the precise execution of a task in order to receive “full” credit. A specific example can be taken from the “posting coins” task in the MABC-2. A child must demonstrate the ability to place coins in a slotted box with the dominant and non-dominant hand on multiple trials. If the child switches hand used mid-trial, then the trial is not considered completely successful regardless of the ability to properly carry out the task. There are also tasks that require multiple attempts to be completed in order to receive full credit. An example noted above is the catching and aiming portion of the MABC-2. The nature of these tasks makes it difficult to discern if poor performance is due to a true motor impairment or compromised by cognitive or attention difficulties leading to poor comprehension of what is being asked [5, 20].

#### Bruininks–Oseretsky Test of Motor Proficiency-2

The BOT-2 provides evaluation from 4 to 21 years of age, which is the widest age range in comparison to the other assessments in this review. The test is made up of four motor composites: fine motor control which evaluates motor skills involving coordination of the distal musculature of the hands and fingers; manual coordination which includes motor skills involving control and coordination of the arms and hands, particularly for object manipulation; body coordination which evaluates control and coordination of the large musculature used in posture and balance; and strength and agility which evaluates aspects of fitness and coordination in casual play, competitive sports, and physical activity. The strength and agility section is unique to the BOT-2 in comparison to the other assessments in this review, and examples of tasks in this category include running, push-ups, hopping on one leg, and sit-ups. Scoring is time and detail intensive and varies per item ranging from a 2- to 13-point scale. Similar to the MABC-2, each item raw score reflects not only the child’s ability to complete a task but might also include the number of correct responses, the number of seconds an activity is sustained, or specific directions provided [21]. However, the BOT-2 does have a scoring mechanism that gives points based off varying degrees of completion. For example, for the item “copying a circle”, the raw score is the sum of scores for “basic shape,” “closure,” “edges,” and “overall size” [21]. Although the points can be obtained with varying degrees of completion, an incomplete drawing will yield a lower score than a complete drawing. In regard to the psychometric properties of the exam, the normative sample of people 4–21 years of age included children with attention-deficit hyperactivity disorder (ADHD), emotional and behavioral disturbance, specific learning disability, mental retardation, developmental delay, and speech and language impairment. In addition, data was also collected on three small clinical samples: developmental coordination disorder, mild to moderate mental retardation, and high functioning ASD/Asperger’s disorder [22]. Inter-rater and test-retest reliability has been shown as well as construct and concurrent validity [21]. In review of the literature, the BOT-2 has had minimal use in children with genetic disorders that confer a high risk for ASD.

As noted in the psychometric properties, the BOT-2 did include a small population of individuals with high-functioning ASD/Asperger’s disorder, making it unique among the other assessments. The BOT-2 also shows particular strengths in ensuring the child can better understand the required motor tasks. Examiners are encouraged to tailor instructions to the needs of an examinee, using verbal directions, physical demonstrations, and photographs provided with the toolkit. The photos supplement verbal instructions which better accommodate children with variable cognitive and behavioral functioning [22, 23]. The BOT-2 also offers multifaceted scoring on certain tasks and allows for a wider range of motor function to be quantified, as most examinees will achieve some success on the task. As noted above, when drawing shapes on the BOT-2, scores are given for basic shape, closure, edges, orientation, overlap, and overall size [22, 23]. Limitations of the BOT-2 are mainly due to the complexity of the tasks and the difficulty this can pose for a child with ASD even with the support of demonstrations. An example from the BOT-2 is the balance task which requires a child to stand onto a balance beam and then attempt to balance on one leg [23]. In some components of the BOT-2 such as manual dexterity, the trials have to be completed in a time-sensitive manner. Additionally, the BOT-2 takes approximately 45 to 60 min to complete and the administration in young children might require two sessions versus one session for an older individual [23–25]. The time-sensitive tasks and duration of examination could be difficult for children with ASD who suffer from co-morbidities such as behavioral dysregulation and ADHD. Although not covered in this review, it should be noted that there is a BOT-2 short form which provides an index of general motor proficiency and is designed to be administered in 15–20 min [24].

#### Physical and Neurological Examination for Soft Signs

The PANESS provides evaluation from 4 to 15 years of age. The test was designed to evaluate deficits of fine and gross motor by testing gait, balance, and aim as well as the presence of “soft neurological signs” such as overflow movements from one body part to another during timed, rapid, repetitive motor tasks and impersistence during stressed gait and oral motor tasks [26, 27]. The PANESS also tests stereognosis and graphesthesia such as having a child close their eyes and identify a number traced in their palm or an object placed in their hand. Items on the PANESS are scored as “1” performed correctly, “2” performed not well, “3” performed poorly or after repeated instruction and demonstration, “4” unsuccessful even after repeated demonstrations, or “9” not done/not ascertained [27]. The administration time is 15–20 min in entirety, which makes the PANESS the shortest assessment in comparison to the other five in this review. In regard to the psychometric properties, it has been reported in the literature that the PANESS should be interpreted with caution [28]. In a small study, the original PANESS had good test-retest results [29], but other studies that have sought to confirm reliability have used revised versions of the PANESS [30]. The revised version was completed due to concerns for items such as the string test and stereognosis being ambiguous and unreliable [27].

One of the strengths of the PANESS is that it was created by a neurologist to evaluate soft signs in gross and fine motor function that can be seen in children with psychiatric and neurologic disorders. The praxis and impersistence tasks assess important motor domains that have been implicated in children with ASD [31]. Neurologic soft signs, praxis, and impersistence are domains that are not evaluated in the other assessments reviewed. The PANESS is also a good tool to quickly test motor function in children with ASD due to the short test duration time. Limitations of the PANESS include the subjectivity of scoring for some of the tasks that has generated questions of reliability in this measure. Also, children with moderate to severe intellectual disability would show difficulty in understanding tasks included in the graphesthesia and stereognosis portions of the assessment.

## Discussion

These six motor assessments have allowed clinicians and researchers gain an initial understanding of the various motor abnormalities that manifest in children with ASD. These direct standardized assessments of motor function move beyond the early indirect clinical observations of motor abnormalities that have been described in ASD, and provide quantification of motor ability that is sufficient to compare and contrast to typically developing children. However, there remain significant gaps in our ability to evaluate motor function in children with ASD, given the heterogeneity encountered, and these gaps are rooted in the individual and global limitations of these assessments.

Identification of these gaps is hoped to spur further refinements in this important area of evaluation.

The first global limitation of all of these assessments is the absence of children with ASD in the normative sample. The normative data is derived from typical development, and therefore, the reliability and validity of the measures have not been well established in a large age range of children with ASD. The BOT-2 attempted to address this limitation by including data on a small clinical samples of ASD/Asperger’s disorder; however, the ASD/Asperger group included a small sample size (*n* = 45) distributed across an age range of more than 15 years [21, 32]. Additionally, anticipated co-morbidities in ASD such as anxiety, oppositional behavior, and attention-deficit hyperactivity disorder (ADHD) are expected to potentially impact a child’s performance on a motor task [33]. Although these standardized assessments achieve the goal of differentiating typical from atypical motor development, given the heterogeneity of motor impairments observed in ASD, it would be beneficial to have normative data from a wide range of individuals with ASD. Utilizing these direct assessments to evaluate a large cohort of children with ASD could provide valuable information on creating motor assessments that are specifically tailored to differentiate motor impairments between individuals with ASD with varying cognitive and behavioral abilities. In addition, motor assessments that are reliable and valid for individuals with ASD can aid in differentiating motor impairments that manifest in ASD versus other neurodevelopmental disorders (NDDs). These data can improve diagnostic classification of motor impairments associated with specific NDDs.

In considering the second limitation of all the assessments, the separation of motor function into two domains, motor abilities and motor patterns, is lacking in the reviewed measures. Clinically, we define motor ability as the child’s ability to complete a task and motor pattern as the way in which the child completes a task or the qualitative nature of the movement. For example, all of these assessments evaluate gait in the gross motor category. The child receives credit if they are able to walk a certain amount of steps or a defined distance. However, if the child demonstrates asymmetric arm swing and an abnormally wide base of support, this is not captured in the scoring mechanism of the assessment. Another example is the threading beads task in the MSEL. The score is based on the outcome performance of the child’s ability to thread the beads. However, the pattern in which the child reaches and grasps the bead and the number of corrections or smoothness of the movement used to insert the thread into the bead is not assessed. These movement patterns are of extreme importance because they may shed light on abnormal neurobiological domains that affect motor abilities in children with ASD. For example, if a child’s gait is wide based and clumsy, then one might consider a disruption in cerebellar circuitry. Alternatively, if there is asymmetric arm swing or difficulty in initiation of a movement then the striatal networks might be implicated. Stratifying motor function into neurobiological domains can be the first step in better understanding the underlying mechanisms that affect motor function in ASD. It can also allow for a better understanding of the source of a child’s abnormal motor function and the most effective method of intervention to improve motor outcomes.

Such information is also likely to demonstrate stronger associations with the severity of core features of ASD. Lastly, none of the assessments include measurement of tone. There are currently no validated standardized assessments that measure tone in infants and children, and hypo- and hypertonia can affect both motor abilities and motor patterns. Hypotonia is particularly prevalent in ASD and likely contributes to delayed achievement of motor milestones and abnormal quality of motor patterns. In addition to evaluating motor function within neurobiological domains, it is also important to consider how hypotonia can affect a child’s gait, grasp, and the ability to engage in a task. A child with hypotonia may take longer to prepare and execute a movement which can affect many of the timed portions of standardized assessments. Similarly, a child might be able to reach for an object but the trajectory and nature of the movement might be affected by low tone. As noted above, the motor patterns or qualitative nature of movement might not be captured in current standardized assessments of motor.

Hypotonia is also common and might be the first sign of atypical development in children with genetic syndromes that confer a high risk for ASD. In Table 1, we included studies that have utilized these standardized assessments to evaluate children with genetic syndromes. In the last decade, there have been rapid developments in the identification of genetic risk factors for ASD that better define the underlying mechanisms of the disorder [8]. These genetic syndromes often present with prominent motor delays prior to core symptoms of ASD. Additionally, it has been hypothesized that these motor abnormalities might lead to more prominent social communication difficulties. Dup15q syndrome (Duplication of Chromosome 15q11.2-q13.1) is an example of a genetic disorder that confers a high risk for ASD and ID and often presents in early infancy with hypotonia and global motor delays [34]. In a recent study, it was shown that children with Dup15q syndrome meet criteria for ASD diagnosis but showed strength in social interest and responsiveness. It was hypothesized that perhaps there is an underlying social motivation but that their profound motor delays interfered with social interaction [9]. If these children are not able to sustain head control or are delayed in their ambulation, then their exploration of the environment and engagement with peers is subsequently affected. These motor impairments can also compound later social impairments in ASD due to children potentially not being able to engage in team sports or being left out of social activities that require agile motor responses. This study in Dup15q syndrome used the MSEL for assessment of motor skills; however, as we monitor these children overtime, it will be important to identify methods of evaluating motor function over a developmental trajectory and in the conjunction with assessments of social motivation and intellectual ability.

### Future directions

Available standardized assessments of motor function facilitate identification of the multiple motor abnormalities in children with ASD but also contain a number of weaknesses. Improved motor assessments are needed for syndromes such as ASD and other neurodevelopmental disorders in order to provide more comprehensive and quantitative “phenotypes,” which provide valuable information to better define the neural underpinnings of motor abnormalities, design intervention targets, and monitor response to intervention. However, with awareness of the limitations of these assessments, there is a growing need for the development of more refined quantitative and objective measures of motor function. Quantitative measures that provide qualitative information such as the nature of gait, posture, and upper extremity trajectory during a task can begin to uncover aberrant underlying neural systems that affect motor function. In addition, methods that do not require cognitively complex tasks can provide evaluation for children with ASD and varying levels of intellectual and behavioral function. It might also be considered that even with a standardized assessment that includes a large normative sample of children with ASD, given the heterogeneity in the disorder, it would be beneficial to utilize both quantitative and standardized assessments in order to capture individual variability in motor function.

The advent of newer quantitative methods of assessing motor function include the use of kinetic and kinematic analysis to quantify specific spatiotemporal variables of motor function such as gait and upper extremity movements. Kinetics is the study of forces that cause motion such as torque, gravity, and friction, and kinematics is the study of movement such as displacement in time and velocity. An additional method utilized is motion capture analysis which can capture whole-body movements of a child to create 3D information of motor function. With increased application in recent studies, the meaningfulness of these enhanced qualitative and objective assessments is beginning to emerge.

In a study of children 3–7 years of age with ASD using kinematic analysis of gait, it was found that children with ASD show significant difference in the preparation phase of movement. These children demonstrated increased variability in the time taken to prepare simple point-point movements relative to typically developing controls leading authors to hypothesize that these findings could support differences in visual processing and visual-motor integration [35]. The use of gait analysis systems that utilize foot pressure variables have shown that children with ASD have wider step width, reduced step rate (cadence), and increased variability in step length [36, 37]. These findings have led researchers to conclude that disruptions in cerebellar and fronto-striatal basal ganglia function are the reason for abnormal movement in ASD [37]. Components of upper extremity action tasks also seem to differentiate ASD from other groups, including horizontal arm movements, reaching-and-grasping, and smoothness and coordination of movement [38, 39]. Kinematic analysis in reach-to-grasp movement is a way to measure spatial and temporal parameters of the upper extremity and has been analyzed in individuals with known motor disorders and underlying intracranial abnormalities such as cerebral palsy, and Parkinson’s disease [40]. The kinematic upper extremity variables generally used are movement time and normalized jerk score which display the performance of motor smoothness and coordination [40, 41]. Motion capture analysis evaluating reach-to-grasp task in children with ASD showed increased movement time, movement unit, and normalized jerk score. This indicated that children with ASD require additional corrective sub movements and poor smoothness in movement execution processes [40]. The same reach-to-grasp task was also completed with and without visual feedback, and children with ASD showed larger movement unit and normalized jerk score compared to controls when no visual feedback was given. Similar findings have been noted in individuals with cerebellar dysfunction who require visual feedback to enhance the accuracy of the reaching movement [40].

These quantitative tools have provided evaluation of new variables of motor function in ASD that can begin to be associated with underlying neurobiological domains. However, many of these studies include small sample sizes and evaluate older and higher functioning children providing limited information on the trajectory of motor function over time in a heterogeneous population of children with ASD. Moving forward, quantitative measures and tasks requiring minimal cognitive ability should be used to evaluate children with ASD starting from infancy through adulthood and with varying cognitive abilities. These studies will enable us to better understand the stability of motor problems experienced by children with ASD which will in turn more closely guide timing of screening and development of intervention protocols.

## Conclusions

The assessment of early motor development is important to clinicians and researchers. Motor abnormalities are pervasive in ASD, are often the first sign of atypical development, and are intrinsically linked to other developmental domains. Motor development is clearly observable and can be measured overtime. There are current interventions that allow for modification and improvement of motor abnormalities, and in turn likely improvement of overall function [42]. For these reasons, it is imperative that specific and subtle motor abnormalities are identified early in children at high risk for and with a diagnosis of ASD. This in turn can aid in developing evidence-based motor interventions that target key impairments in ASD. Standardized assessments have been valuable in identifying some core motor deficits in ASD but they often fail to capture the variability in motor patterns which can provide valuable insight into the underlying mechanisms affecting motor function. Objective and quantitative measures of motor function and assessments of domains such as tone should be a priority for future research. With such efforts, we may begin to stratify the heterogeneity in motor function across the spectrum of ASD and genetic conditions associated with ASD, perhaps revealing unique endophenotypes of motor function and developing more targeted interventions that ultimately provide improvement in multiple developmental domains in individuals with ASD.

## Abbreviations

ADHD: Attention-deficit hyperactivity disorder
ASD: Autism spectrum disorders
Bayley-III: Bayley Scales of Infant and Toddler Development-III
BOT-2: Bruininks–Oseretsky Test of Motor Proficiency-2
MABC-2: Movement Assessment Battery for Children-2
MSEL: Mullen Scales of Early Learning
NDDs: Neurodevelopmental disorders
PANESS: Physical and Neurological Examination for Soft Signs
PDMS-2: Peabody Developmental Motor Scale-2
